# Vaccine hesitancy among health paraprofessionals: A mixed methods study

**DOI:** 10.1371/journal.pone.0312708

**Published:** 2025-01-07

**Authors:** Madeline Hergott, Michael Andreski, John Rovers

**Affiliations:** College of Pharmacy & Health Sciences, Drake University, Des Moines, Iowa, United States of America; Yarmouk University, JORDAN

## Abstract

**Background:**

The World Health Organization (WHO) defines vaccine hesitancy as “a delay in acceptance or refusal of vaccines despite availability of vaccination services”. Vaccine hesitancy has also been declared a top threat to global health. Some employers imposed vaccine mandates during the Covid-19 pandemic resulting in health care employees resigning or being fired rather than receive a vaccine. Healthcare paraprofessionals such as certified nursing assistants, dietary and home health aides are among the most patient facing of all health care providers. Their beliefs and attitudes about vaccines are critical to how they communicate about vaccines with their patients.

**Objective:**

The objective of this project was to survey health care paraprofessionals to explore their thoughts and opinions about vaccines in general, and Covid-19 vaccines specifically.

**Methods:**

This was a 25 question, mixed methods, cross sectional email survey. Subjects were recruited from the mailing list of a non-profit organization in the Midwest. This organization is dedicated to bringing a face and a voice to healthcare paraprofessionals engaged in direct patient care.

**Results:**

Most respondents were reasonably well informed about vaccines; had received one or more doses of indicated vaccines; used credible resources to learn about vaccines and believed physicians and pharmacists were the most trusted information sources. Qualitative results indicated that respondents expressed support for vaccines but that the support was often qualified in that a respondent may have had both pro and anti-vaccine opinions in the same response. They also expressed that communications about vaccines were often problematic. Additional vaccine-related continuing professional development for healthcare paraprofessionals appears to be indicated.

**Conclusions:**

Although attitudes towards vaccines were generally positive, respondents had concerns about the quality of vaccine information. Additional vaccine-related continuing professional development for healthcare paraprofessionals appears to be indicated.

## Introduction

According to the World Health Organization (WHO), vaccine hesitancy may be defined as, “a delay in acceptance or refusal of vaccines despite availability of vaccination services. Vaccine hesitancy is complex and context specific, varying across time, place, and vaccines. It is influenced by factors such as complacency, convenience, and confidence” [1]. The WHO has also declared vaccine hesitancy as a top threat to global health [2].

The onset of the Covid-19 pandemic in 2019 and the speed with which effective vaccines became available have caused a greatly enhanced public health focus on vaccines, and more specifically, vaccine hesitancy. Covid-19 vaccines first became available in the US in December 2020 [3]. Uptake of the vaccines was rapid at first, but by April 2021, had begun to slow. By September 2021, an estimated 70% of healthcare workers were fully vaccinated. This is lower than what is needed to keep both patients and other healthcare workers safe from the virus.

The highest uptake was seen by workers in pediatric hospitals (77%) while the lowest uptake was found in rural hospitals (65.1%). Prior to the availability of Covid-19 vaccines, Kose and colleagues surveyed 1138 healthcare personnel. Only 69% of respondents stated they could be vaccinated (51% of physicians, 5% of nurse/midwives, 72% of medical/nursing students, 61% of other personnel). Eleven percent did not want a Covid -19 vaccine, while 20% were unsure. Those who were younger, male, and previously vaccinated against influenza were more likely to accept a vaccine if one were available [4].

One response to both the pandemic and the availability of vaccines was vaccine mandates. Such mandates usually required an employee to provide proof to their employer that they had been vaccinated. Although generally not supported by most courts or governments, private employers are permitted to require their employees to be vaccinated as a condition of employment [5].

Unfortunately, one of the consequences of such private employer mandates was that some employees would rather quit their jobs or be fired than comply with the requirement to be vaccinated [3–6]. In one large Health Maintenance Organization, 2200 of 240,000 employees were placed on leave due to refusing to be vaccinated, while at a large hospital in Texas, 150 employees were fired or chose to resign after an employer mandate [6, 7]. The loss of critical employees at a time when the healthcare system was near the breaking point due to Covid-19 both creating a large influx of critically ill patients and a simultaneous large number of employees needing sick leave themselves resulted in an untenable situation for many health systems [8].

It is clear that a priority for public health researchers is gaining an understanding of why some healthcare employees may refuse a vaccine [3]. Unless we understand the reasons for vaccine hesitancy, it is unlikely we will identify useful responses to promote vaccinations.

A number of previous surveys that explore vaccine hesitancy have been published. Opel and colleagues used the Theory of Planned Behavior to create a questionnaire, but their instrument was designed to study vaccine hesitancy in parents [9, 10]. Dubé and colleagues developed an explanatory model based on a combination of the Theory of Planned Behavior and the Health Belief Model [11].

Other authors discuss various methods by which individuals process risk, and how such processes influence the decision to vaccinate or not [2]. Lin and colleagues offer that general factors explaining vaccine hesitancy include the attributes of both the vaccine and the disease (safety, barriers to access, disease severity), characteristics of the healthcare provider (younger female providers, pediatricians more trustworthy, extensive provider clinical experience and knowledge of disease, and vaccine if provider is vaccinated), patient characteristics (low socio-economic status, race, co-morbidities), and system factors (vaccine guidelines, confidence in vaccine, clear official recommendations, source of information, practice setting) [12]. Nagar and colleagues explored religious objections to vaccines and found that White evangelicals, Black Protestants, and Hispanic Catholics were likely to be the most vaccine hesitant. Religious reasons given include vaccine misinformation (inclusion of fetal tissue in vaccines), conspiracy theories that nefarious actors seek to harm the virtuous, and experiences of prior racism in healthcare settings [13].

A major gap in the literature about vaccine hesitancy is to explore the opinions of healthcare paraprofessionals such as nursing aides, dietary aides, home health aides, etc. These paraprofessionals are often the most patient facing of healthcare providers. Ensuring that these providers have a solid knowledge base and positive attitude towards vaccines is a valuable tool within the public health armamentarium as these providers interact with their patients.

## Objective

The objective of this project was to survey health care paraprofessionals to explore their thoughts and opinions about vaccines in general, and Covid-19 vaccines specifically.

## Methods

### Survey

This was a 25 question, mixed methods, cross sectional email survey. The survey for this study was developed using a combination of two health behavior theories (Theory of Planned Behavior and The Health Belief Model [14] as well as data from the aforementioned literature that explains vaccine hesitancy.

A 25-question survey was developed. Forced choice questions were asked about: respondent demographics; prior knowledge of vaccines; vaccination history for themselves and family/friends; reasons to be or not be vaccinated; benefits of vaccination; preferred and trusted information sources for vaccine information; who should set vaccination policies; voter registration.

Finally, one open ended question was included where respondents were able to provide additional thoughts and experiences about vaccines.

Prior to distribution, we pilot tested the survey for clarity and ease of completion with a convenience sample of healthcare workers ineligible to participate in the project. Only minor changes were made.

The survey was put into the field on January 18, 2023, and a reminder sent on February 14, 2023. The survey was closed out on February 28, 2023.

Respondents who opened the survey gave informed consent by reading the statement of informed consent and then clicking the radio button to indicate consent (which took them to the survey) or declining to participate (which ended the survey).

### Sample

Subjects were recruited from the mailing list of a non-profit organization in the Midwest dedicated to bringing a face and a voice to healthcare paraprofessionals engaged in direct patient care. This organization does not have members in the traditional sense of the term. Rather, most of its mailing list is taken from a state direct care worker registry.

Inclusion criteria were to have a valid email address, aged 18 or older, and able to read and write either English or Spanish. Exclusion was anyone who did not meet these criteria.

### Data collection

A Qualtrics survey in English and Spanish was sent as an email to all addresses in the organization’s database. The survey was kept in the field for a total two weeks at which point a reminder email was sent out. The survey was closed two weeks after the reminder email was sent.

### Data analysis

Quantitative data were analyzed by tabulating the quantitative results. We also attempted to perform a multivariable logistic regression analysis to assess any relationships between demographic or other data and answers related to forced choice questions about vaccines.

Qualitative data were analyzed using Thematic Analysis. Two authors (MH and JR) each created a code book independently and performed manual open coding on the transcripts of the open-ended question at the end of the survey. The open coding was grounded in interpretive phenomenology since the analysis required interpretation of the lived experiences of the respondents and was not dependent upon a preexisting theoretical preconception. Interpretive phenomenology has been shown to be valuable in previous studies of respondents’ perspectives [15].

After open coding was completed, all three authors met to review the codes and finalize the code book. Author MA served as a referee when the other authors did not agree on a code. Authors MH and JR then re-coded the data using the revised code book. MA served as referee when the others disagreed on an assigned code. With all authors in agreement on the assignment of codes, MH and JR then independently performed axial coding on the transcripts to identify themes that emerged from the data. MA served again as referee when the other authors did not agree on a theme identified.

## Ethical approval

The study was deemed exempt from full board review by the Drake University Institutional Review Board (IRB Proposal #: 2021–22106). Respondents were able to click a box to indicate willingness to participate in the research. Respondents were also offered the ability to be eligible for a $100 gift card for their participation.

## Results

**Fig 1:**
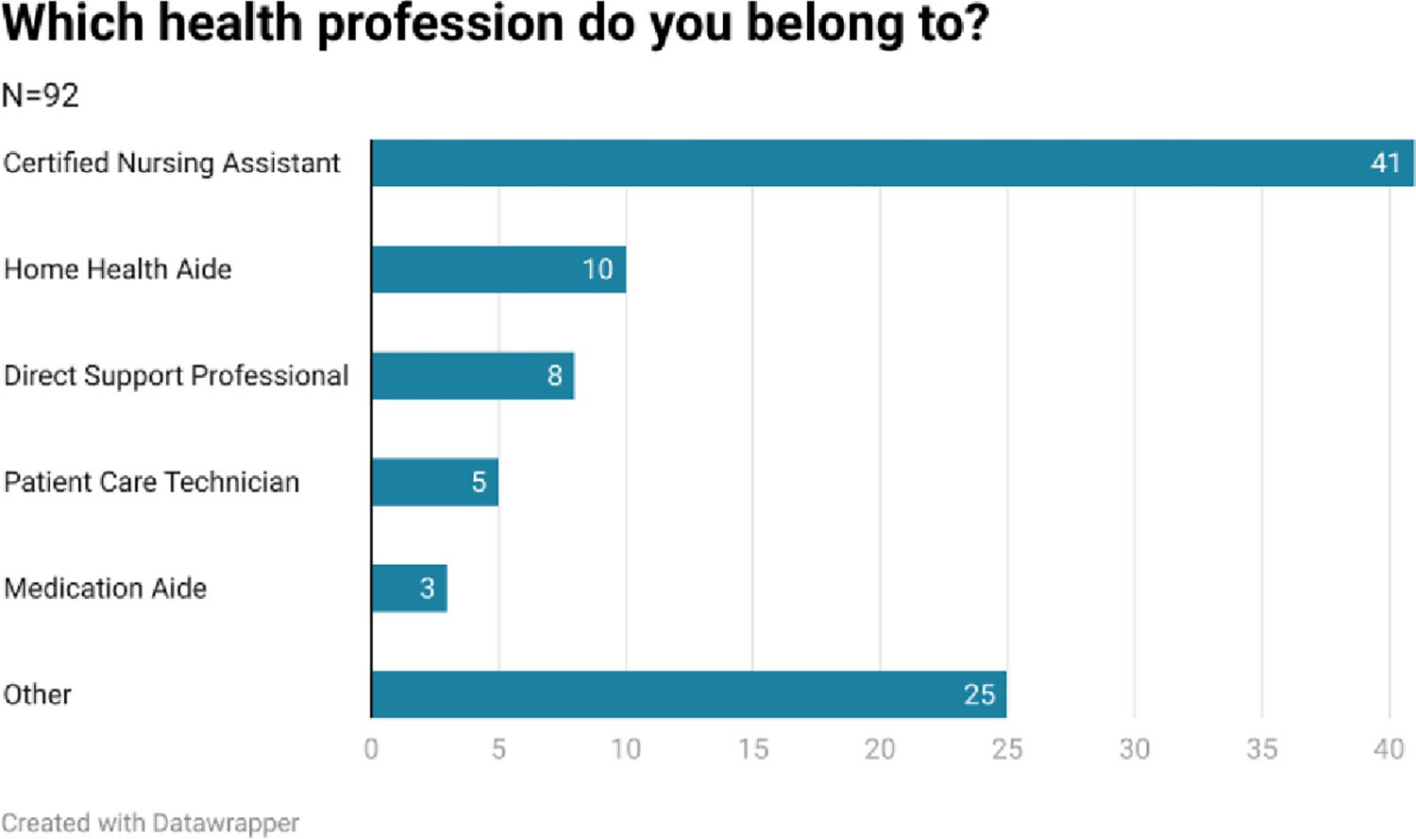


The survey link was initially sent to 34,087 email address followed by 34,846 email addresses after two weeks in the field. The email was opened by 36% of addressees. Click through rates to the survey were 1% both times, resulting in 92 useable responses. As such the overall response rate for useable surveys as a proportion of surveys sent was 0.27%. All responses were in English, none were in Spanish. Respondents’ health professions are shown in Fig 1.

### Quantitative results

**Fig 2:**
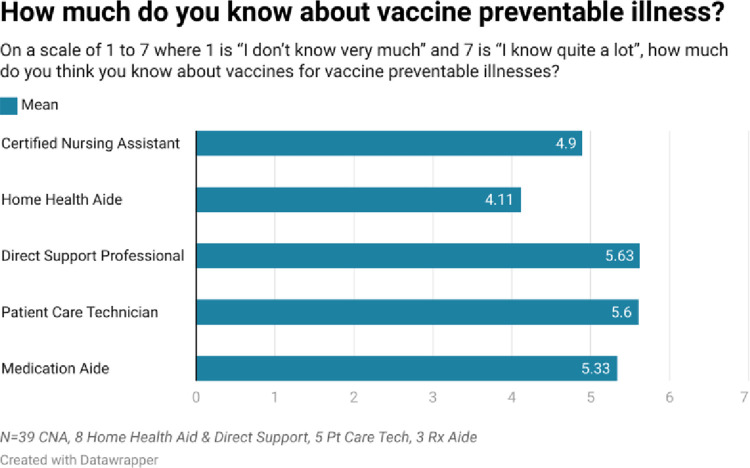


**Fig 3:**
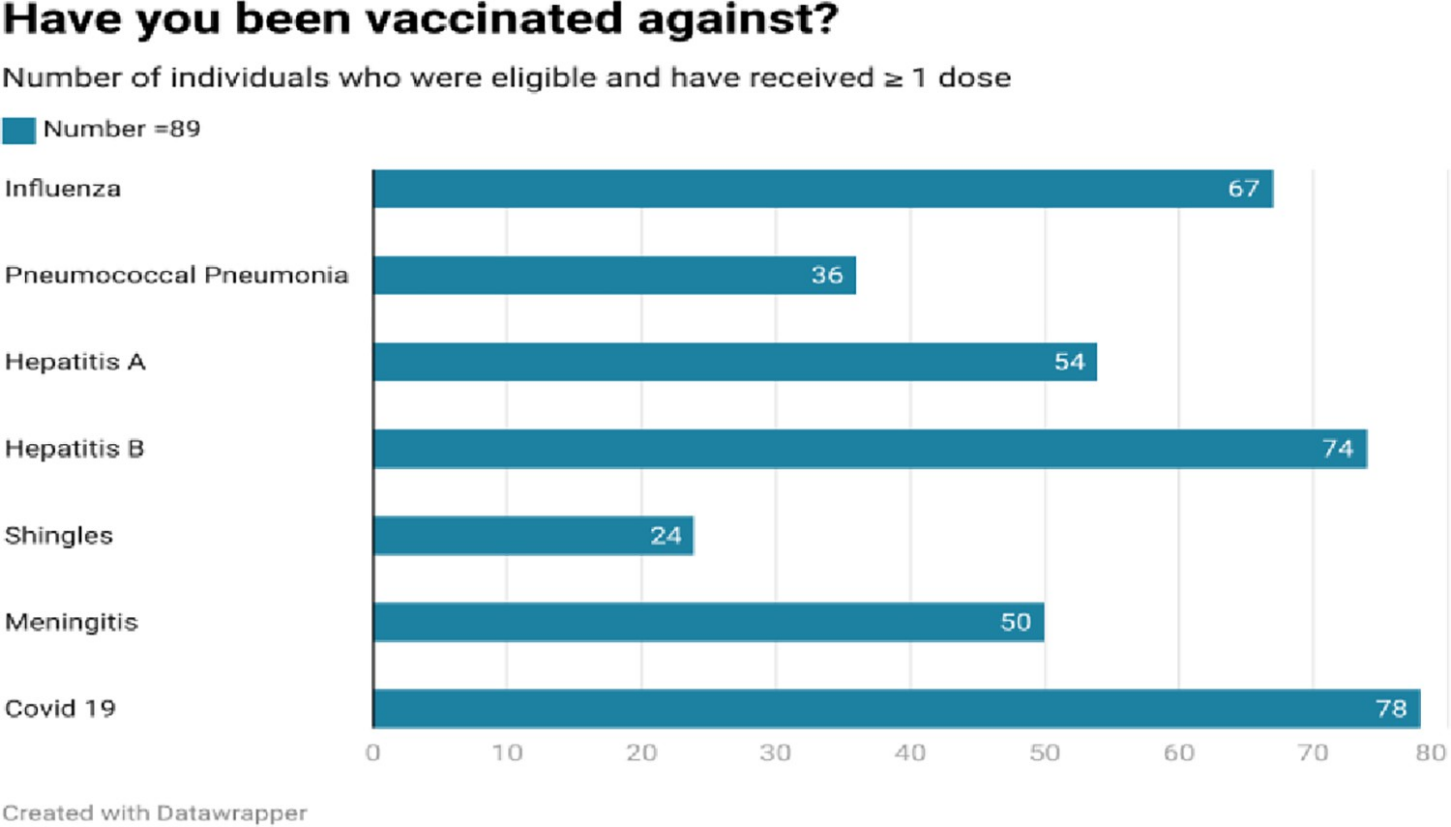


**Fig 4:**
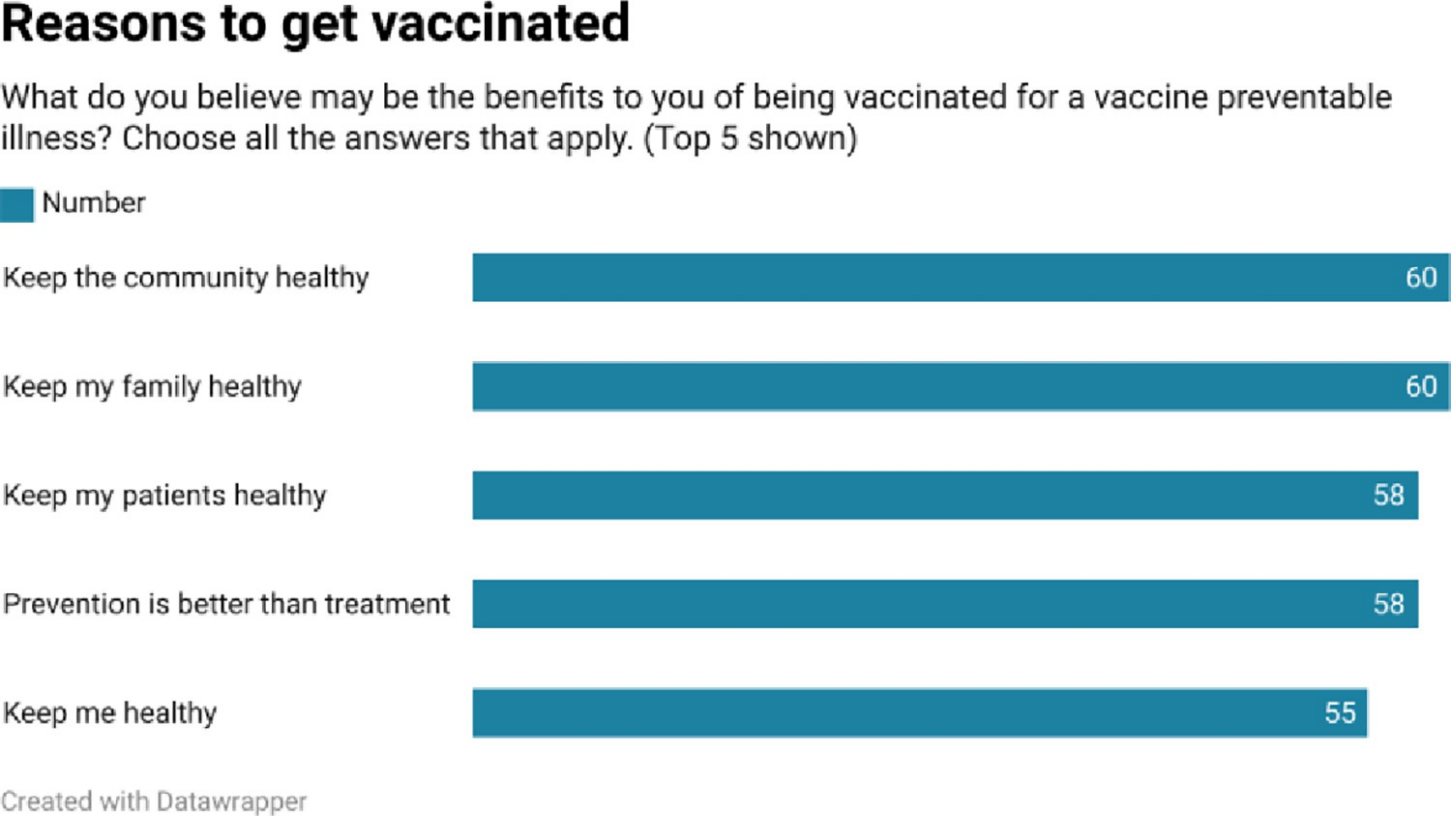


**Fig 5:**
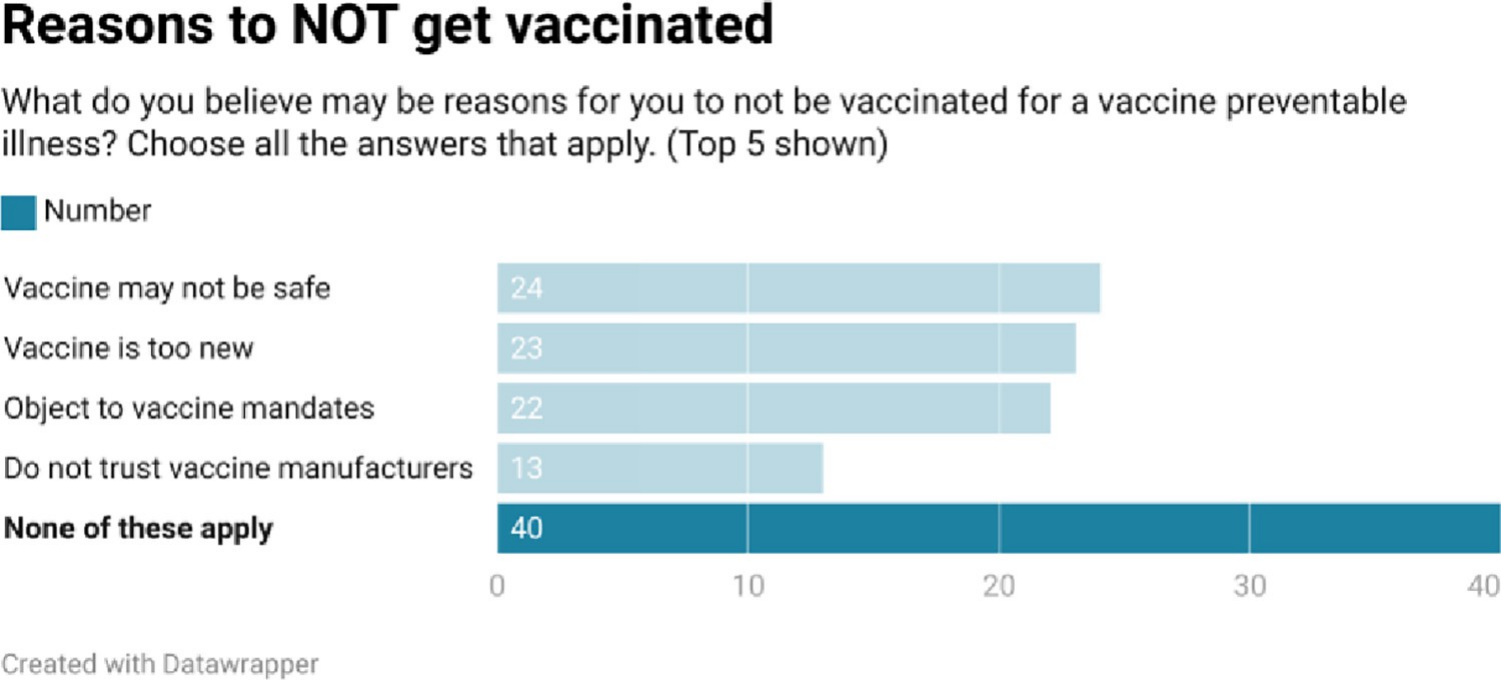


As shown in Figs 2–5, respondents were generally well informed about vaccines, had been vaccinated themselves, and were of the belief that vaccines are beneficial.

**Fig 6:**
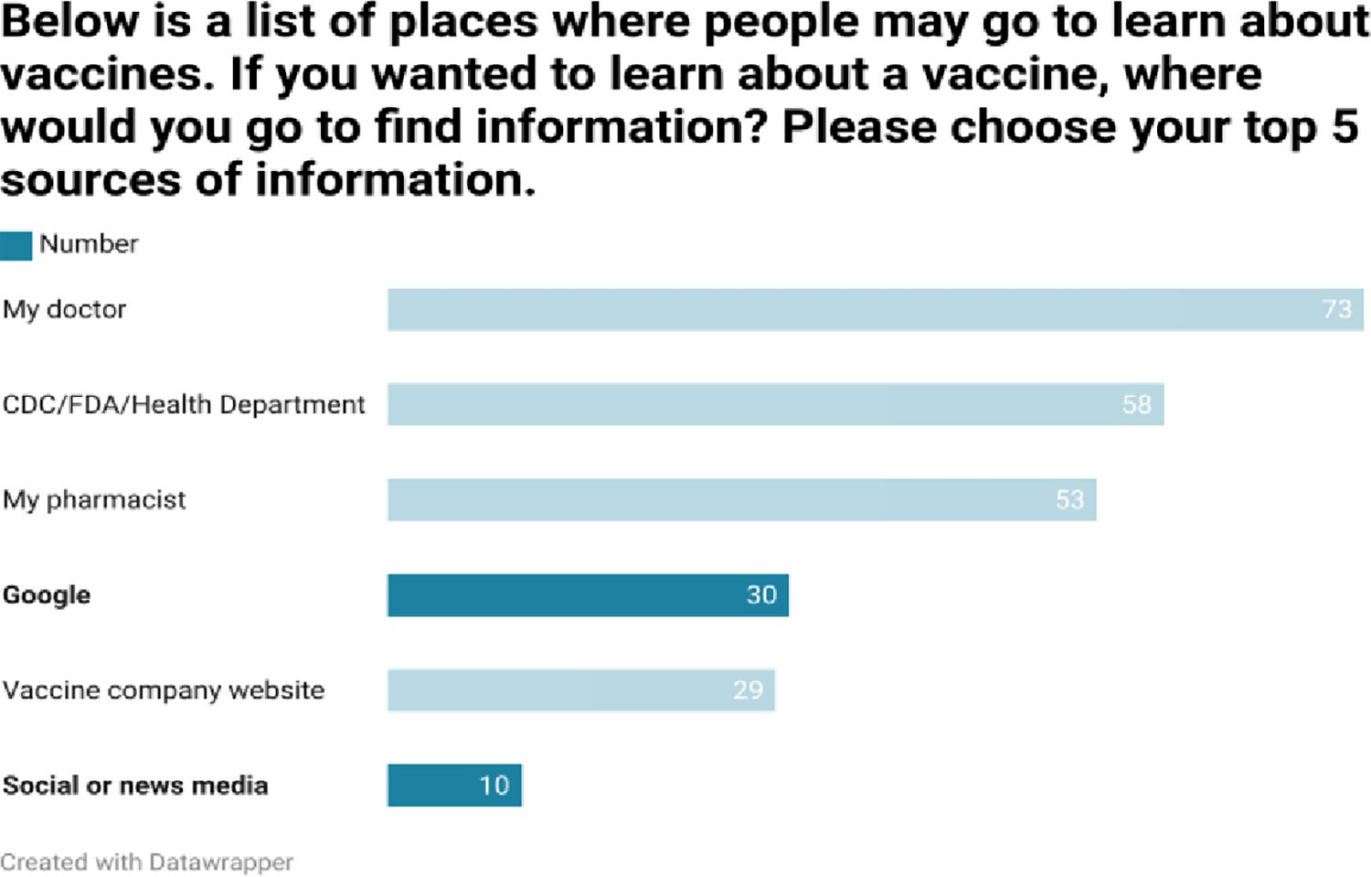


**Fig 7:**
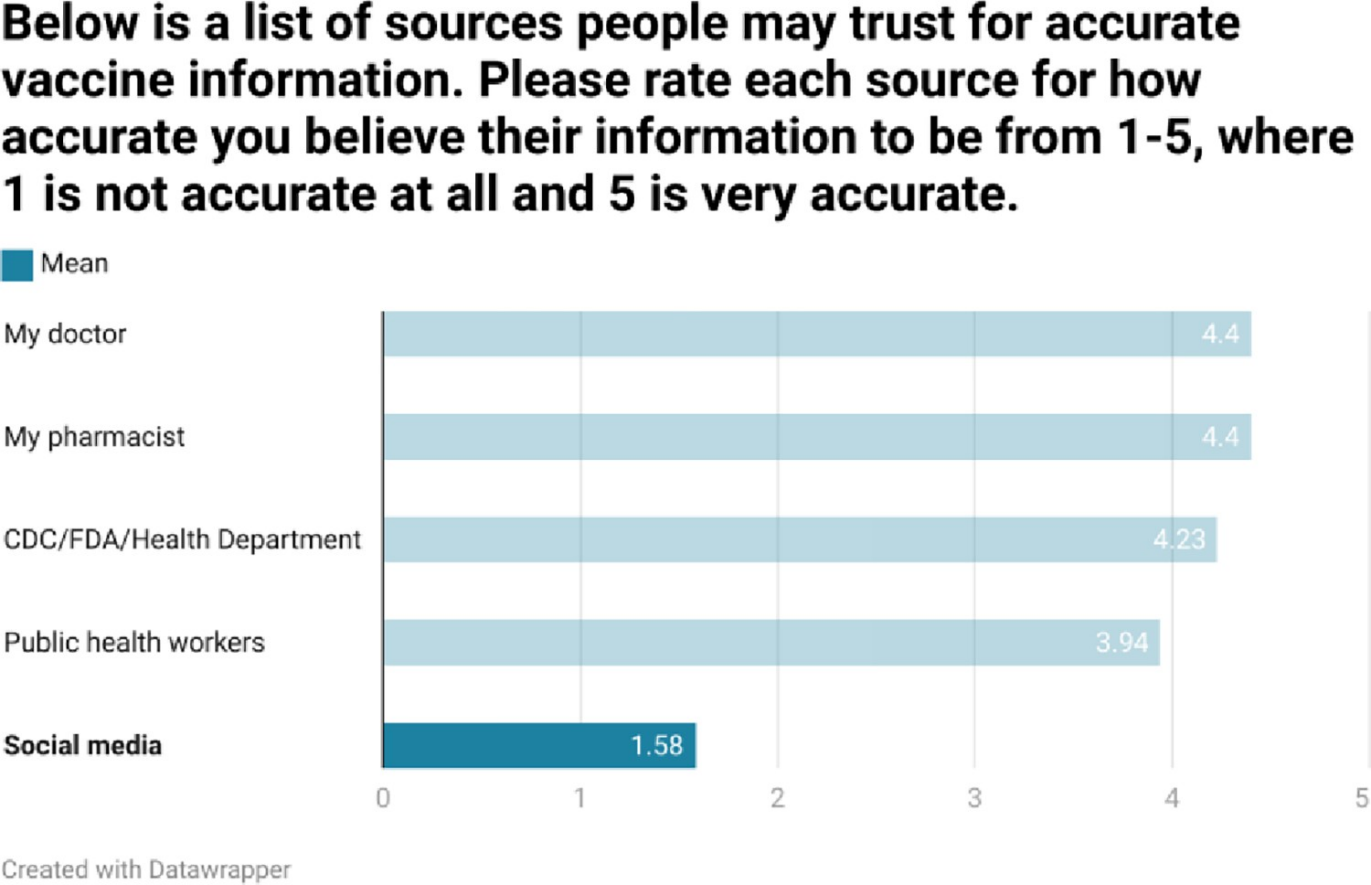


As shown in Figs 6 and 7, although respondents typically used credible sources of vaccine information, Google and social media were used occasionally, although social media was held to be less credible than other sources of information.

Multiple attempts at logistic regression did not reveal any predictive relationships on vaccine opinions or behaviors between any of the variables.

## Qualitative results

**Fig 8:**
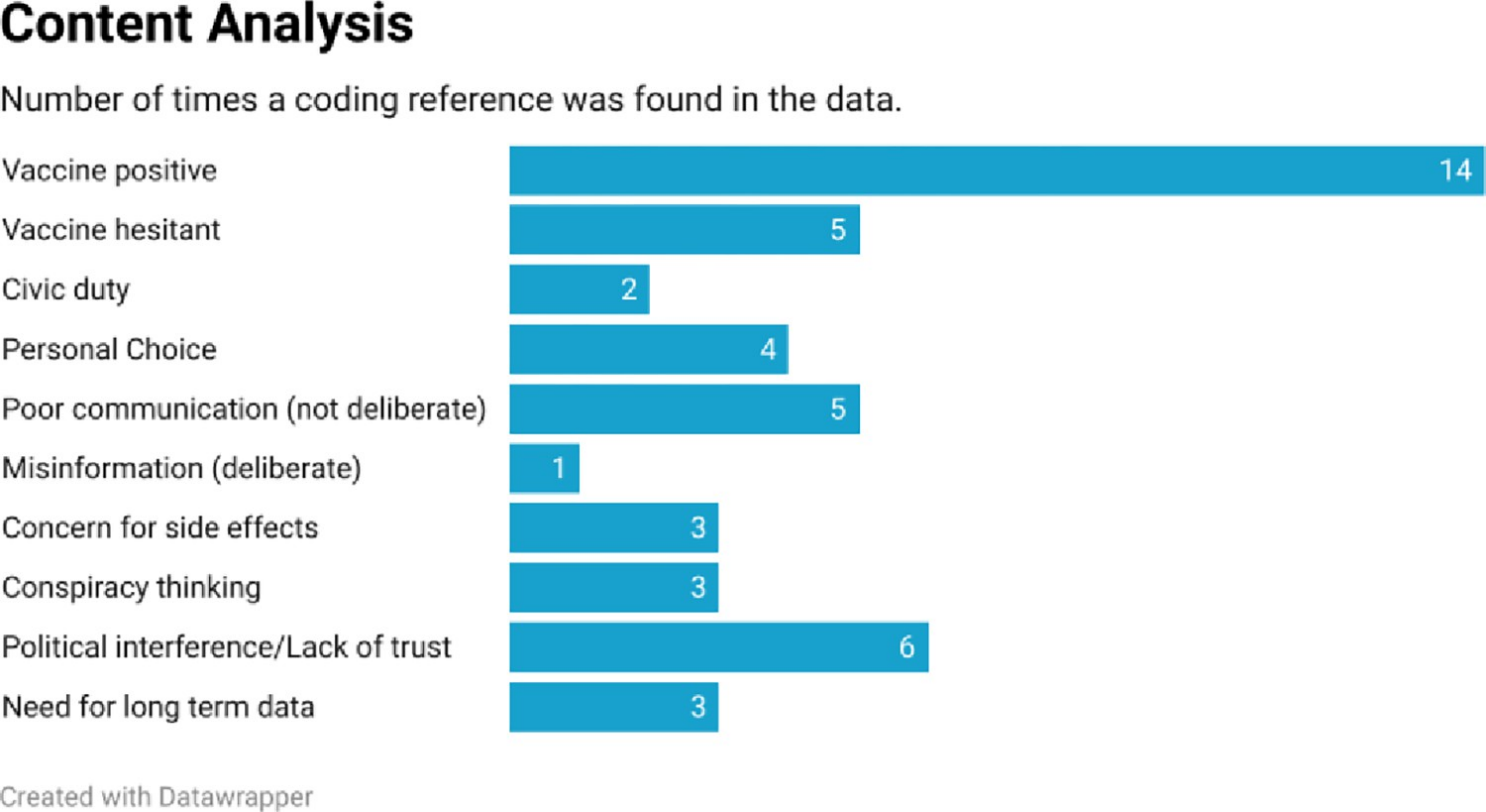


The final code book used in open coding included 10 codes. Using the final codebook, when authors MH and JR coded the transcripts individually, there was a 67.3% agreement between analysts. Author MA served as referee to determine the final assignment of the appropriate code to a fragment of text. Fig 8 shows a content analysis of how many final coding references were found in the transcripts for each code.

There were 26 responses to the open-ended question of, “If there is something you would like to tell us about your thoughts and experiences about vaccines, please write them in the box below.”

Two themes emerged from the qualitative data:

1. Vaccines are generally supported by respondents, but the support was often qualified.
2. Communications are a problem. Only some information is deliberately misleading, but even positive communications are not well done.

Some of the support for vaccines was unqualified:

*I have worked in a covid 19 outbreak before and after vaccines..before vaccines the outbreak was devastating! Of 40 residents, 30 residents got covid as well as most of the staff..we had at least 10 residents pass away…since the vaccines we have been in a outbreak with only 4 residents..the spread of covid since vaccines does not even compare to pre vaccination..the devastation has been far less after vaccines..*

Certified Nursing Assistant

*I have never had a bad experience with an* (sic) *vaccine. I think that everyone should be vaccinated.*

Home Health Aide

More often, support was positive, but qualified. Frequently, respondents expressed both support and hesitation in the same comment. Concerns about safety, efficacy, long-term data, and the motivations of the pharmaceutical industry were also expressed:

*I fully support getting vaccinated against the big diseases like measles, mumps, chickenpox, hepatitis. I don’t support getting rushed vaccines like COVID-19. I feel there was a lot of outside involvement in COVID-19 and there shouldn’t have been.*

Other paraprofessional

Beliefs that pertain to the COVID vaccine are set under different parameters than all the shots we received as children. Those were proven highly contagious diseases. I’ve never gotten the new vax and had COVID maybe once, with just a cough for a couple days and fatigue. So, I saw no urgency or necessity. I’m careful enough to not spread even I am an asymptomatic carrier.

Certified Nursing Assistant

*Almost all vaccines are cool*. *I have all of my childhood and young adult vaccinations*. *I got the two initial doses of COVID from mederna* (sic) *but did not get any boosters bc I’m relying on my immune system which*, *historically*, *has been resilient*. *Same with the flu vaccine*, *plus there’s no guarantee that the vaccine is designed for the flu I will be exposed to*. *I think an important aspect that relates to preventing illness is personal hygiene*, *I wash my hands when needed and use hand sanitizer going on and coming out of buildings if available*.

Direct support professional

I think that surveys like this one make it difficult to properly give my views on vaccines. I’m not anti vaccine, I was very pro vaccine prior to covid and I still am for all vaccines except for the covid vaccine. Surveys like this ask for our opinions on vaccines but make it difficult to answer when we single out one that we aren’t willing to get. The way that this vaccine was pushed on the public and the side effects that we have seen since, make me hesitant to get it. 5 years down the road when we see the long term side effects and pharmaceutical companies have the opportunity to make adjustments, I’d be more willing to get it. People that are part of medical studies get paid very well to do it. If I wanted to participate in drug studies, I would go there to do so, but I am not willing to be a science experiment. I will wait for the long term science to prove to me that it’s worth it. My coworker is very pro covid vaccine and has it currently and has been extremely sick for over a week now. I had it over the summer and was fine within two days.

Other paraprofessional

I have been very lucky. Vaccinated and boosted, and never got Covid. I’ve had many friends that have had the same vaccinations and has had it at least 3–4 times. One had it so bad she was hospitalized. Works for some??!!!

Certified Nursing Assistant

Respondents often expressed concern about how vaccine information is communicated. The role of the internet and political interference was concerning to some.

*With the advanced science and technology we have today, We have the ability to treat most of the illnesses that vaccines are said to protect us from. No vaccine should be administered to anyone outside of a research facility until we know long-term effects over at least a 20-year span. Vaccines should only be made and distributed by those licensed in the medical field. Those working in politics, should have no say or effect on vaccinations in any way shape or form.*

Other paraprofessional

As an employer, I find a lot of younger staff listen to others or think they have off beat and secret information from the good ole internet. I have taken every vaccine offered since I was a kid in 1958. I have suffered no ill effects. I trust vaccines. I blame too much internet and self appointed "experts" as the idiots that decry vaccines. I think they have been hogwashed by the same system they blame… ..

Other paraprofessional

Should be communicated better versus telling people what needs to be done, talking to people instead of at them changes a lot of rebellious behavior!

Certified Nursing Assistant

I feel that having mandates help protect somewhat but feel as though illnesses are made by the government to depopulate but then come up with a vaccine that isn’t 100 percent and still causes far too many problems before the right one comes out for a vaccine. All about the 1% and what they choose for us peeons (sic)… .

Patient Care Technician

I believe vaccines are a GOOD thing. I have been taking vaccines since I was little. I am 65 and I grew up in a time when vaccines were trusted. The INTERNET has caused EVERYONE TO BELIEVE THEY ARE AN EXPERT WHEN IT COMES TO VACCINES. People have lost sight of common sense and the benefit of a Medical Education and Medical Professionals advice when it comes to vaccines. I BLAME THE INTERNET AND CONSPIRACY THEORIES AND OUR CRAZY SOCIETY THESE DAYS. Vaccines work period. Thanks.

Direct support professional

Although comments that were highly critical of vaccines were uncommon, several respondents expressed extreme skepticism about available vaccine information. Some of the opinions they expressed appear to be deeply held, but factually incorrect:

*I have been studying vaccines for 30 years. I have read numerous books and researched them extensively. I’m sorry to say that I know more about them than most including many who others would turn to for answers. I don’t consult them because they only know what they themselves have been told. My sources have been completely unrelated, spaced years apart and everything I’ve learned confirms the other’s findings and then some. The books I’ve read are among the most highly researched and documented books I’ve ever read, backed by many studies. It’s not about fear, it’s about facts, science and understanding the way vaccines are made, what they can do to the body, how they work (and don’t work), how our society has been lead like sheep once again to subject themselves to the greater evil for the sake of profit. Vaccines are not standardized. No other drug is given to a newborn (when studies were done that said they shouldn’t be administered to those under the age of 2 yrs), in the same dose as would be given to a 5 yr old. It’s NEVER, done. Dosage is metered by weight and age. Not so with vaccines. Parents aren’t told the dangers of administering these toxins to their children if they have contraindications such as central nervous system instabilities, or even a common cold. Parents don’t know, and they should. Also, because the vaccines are not standardized, there are "hot lots" and those aren’t even removed from the market until a certain number of children die. Then, they’re sent overseas to 3rd world countries instead of being destroyed! Most doctors don’t even know these things. The CDC does though. Watch the documentary "Vaxxed" There are 2 actually. Read "A Shot in the Dark." Read "What Every Parent Should Know About Childhood Immunizations". Read the periodical "Mother Earth"—the issues that contain articles on the World Symposium on Immunization. Other countries do not immunize or immunize much later than the US. We have THOUSANDS of children injured or killed by immunizations every year. It’s a deadly game we play assaulting the human body with toxins in the name of profit and "protection." It’s definitely something that should be extensively researched prior to partaking of and I’d rather take my risks with the diseases and my natural immunity that the than vaccinate. It’s not enough for us. It’s far too much for them. This is no conspiracty* (sic) *theory; There is an organized corruption behind most of the vaccination industry. A medical mafia if you will. Certainly not all doctors by any means. In fact I would say the minority. However, the ones involved are the ones in charge and pull the strings keeping the rest in the dark. The blind, leading the blind. It’s shameful. People shouldn’t be so trusting. They should learn to research for themselves. Their children’s lives are at stake as well as their own now. I imagine I sound like a fanatic spouting rhetoric, but I assure you, that’s anything but the truth. Every word here is founded and supported.*

Certified Nursing Assistant

If vaccines are good for the human body please explain to me why over 90% of American’s have died from getting the vaccines and or have had long term health effects that they can never get rid of?

Certified Nursing Assistant

## Discussion

Our quantitative results reveal a population of health paraprofessionals who were reasonably well informed about vaccines (range 4.1/7–5.6/7), and who had themselves largely been vaccinated with vaccines for which they were eligible. With the exceptions of pneumococcal and shingles vaccines, at least 50% of respondents had received ≥ 1 dose of the appropriate vaccine. The most common reasons to be vaccinated reflected respondents’ concerns for the community, their families, and their patients. Protecting themselves from a vaccine preventable illness was less commonly cited as a reason to be vaccinated. When asked a variety of reasons not to be vaccinated, the most common response was none of the reasons offered applied.

Similarly, respondents typically sought vaccine information from credible sources and felt that heath care professionals and public health resources were the most credible sources of vaccine information.

Our qualitative results are largely reflected in our quantitative ones. Statements supportive of vaccines were frequent which is reflected by the high number of respondents who were vaccinated. Reasons to choose not to be vaccinated were less frequent than the reasons respondents chose in favor of vaccination. Self-reports of reasonable vaccine knowledge are supported by the use of credible vaccine resources. Overall, our results suggest most respondents were well informed practitioners, able to choose appropriate information sources, and who had made clinical judgements that vaccines were of benefit to themselves and their communities. Our results appear to be largely positive.

Positive results notwithstanding, it is important to note that much of the positive support was qualified in that a respondent may have had both pro and anti-vaccine opinions within the same response. Concerns about safety, efficacy, long-term data, and the motivations of the pharmaceutical industry were expressed. Although credible sources of vaccine information were the most frequently ones sought out, Google was the fourth most common information resource. Although to a lesser extent, social media were also cited as information sources.

These results suggest that even in a cohort well informed and supportive of vaccinations, there remains a need to provide continuing professional education (CPE). Health care providers with concerns about vaccines are unlikely to be strong vaccine advocates, and without such advocates, the risks of vaccine preventable illness are higher. These concerns are supported by the literature. Hsu and colleagues note that staff in long term care (LTC) settings had the lowest vaccination rates of all frontline care workers [16]. In a study of vaccine knowledge, attitudes, and recommendation practices, Fernandes and colleagues found that 42% of respondents believed there were vaccine hesitant staff employed in their medical practices [17].

Limaye and colleagues found that how a vaccine message is framed and who delivers it may affect the vaccine decision making process [18]. Trusted experts such as physicians, nursing directors, consulting pharmacists, and peers may all be suitable individuals to provide CPE to frontline care workers. Although much of the literature around limiting vaccine hesitancy and providing education concerns communications with patients or parents, it lends itself to be readily adopted to provide vaccine CPE in LTC workers. Most interventions are designed to minimize the attendee’s perception of risk posed by the vaccine.

Simply providing advice and information is often ineffective when speaking with the vaccine hesitant. Weinstein and colleagues state that conversations with the vaccine hesitant be as specific and honest as possible [19]. Interviewers must be honest and specific about what is not known about the vaccine. Vague assurances that vaccines are safe and well tested are generally ineffective.

O’Leary and colleagues state that honest, bilateral communication from a trusted provider may lessen fears about vaccines [20]. They provide Motivational Interviewing (MI) as an example of honest, bilateral communications. Fernandes and colleagues also support the role of MI and note that poor communications by professionals can negatively affect vaccine attitudes. MI is a communications method in which the interviewer listens closely, guides the discussion to engage with the interviewee, clarifies their concerns, motivates change, and promotes autonomous decision making [21].

One example of how MI can be employed is to use Haidt’s Moral Foundations Theory when discussing vaccines [22]. He notes there are six foundations we use when making decisions with moral implications: Care vs. Harm; Fairness vs. Cheating; Loyalty vs. Betrayal; Sanctity vs. Degradation; Authority vs. Subversion; Liberty vs. Oppression.

When speaking with a vaccine hesitant person, Rovers and Thompson advise asking open-ended questions to learn which moral foundations are influencing someone’s decision making. For example, if a person’s moral decision is influenced by concerns of oppression (e.g., vaccine mandates) providing feedback with examples of common limitations of liberty we already comply with (e.g., no smoking areas, not driving drunk) may be effective. If the motivation is care vs. harm, the interviewer can note that getting vaccinated oneself demonstrates caring since vaccines lower the risk of disease transmission. This type of discussion normalizes the use of vaccines and can assuage emotional concerns [23].

Chou and Budenz confirm that emotions play a significant role in the decision to vaccinate [24]. In the LTC setting, CPE should consist of downplaying negative motions such as fear, anxiety, needle phobia, etc. They suggest enhancing self-efficacy by structuring vaccine conversations as concrete, actionable strategies that reduce risk. Advocating that vaccinating is a prosocial activity that benefits the community and “inoculating” staff against antivaccine positions before they are aware of a negative viewpoint may also be helpful. Yang also notes that a person’s emotions can affect pro-vaccine appeals and perceptions of risk [25].

Limaye and colleagues are more skeptical of the role of personal storytelling and appeals to emotion [18]. Instead, they suggest that vaccine promotion education be based in appropriate theoretical constructs that minimize risk and enhance self-efficacy. That said, they also acknowledge that sometimes stories are more compelling than statistics. They also support the use of social media to promote vaccines but note that “pre-bunking” and “inoculation” (posting a positive vaccine position before negative postings emerge) may be more effective than debunking misleading information.

These same authors also describe a massive open-enrollment online course (MOOC) used in vaccine related CPE [26]. The course took advantage of using a peer-to-peer method to create the course. The course was created using both experts and peers to create four modules on vaccinations (vaccine hesitancy, the immune system, communication techniques, and identifying and countering misinformation). The MOOC was found to be successful, and the use of peers allowed for the course to take advantage of their lived experiences with vaccines when teaching others. This approach may be useful in LTC settings with staff working multiple shifts and unable to gather at one time. Hsu and colleagues have also used a peer-to-peer approach using educational materials created by the Centers for Disease Control and Prevention (CDC) [16]. In their study, two to four employees working in LTC facilities tailored the education message to their peers to create CPE vaccine workshops. Using peers to communicate may be especially important if LTC employees share a cultural or linguistic heritage. Peers may be more likely to identify and address workers’ concerns than outside experts.

## Limitations

Several limitations to the project are noted. The response rate to the emailed surveys was seemingly low. The non-profit agency whose mailing list we used suggested that those on the mailing list are over-surveyed and response rates to other surveys have been historically low. However, the email addresses used are not actually a membership list. Rather, they are just names taken mostly from a state registry and not all recipients may have been aware of where the survey came from.

We would also note that 36% of addressees opened the email. The 1% click through rate to the survey link meant that approximately 340 individuals opened the survey. Of these, 92 (~ 27%) completed the survey and 26 (~8%) provided written comments. Previous literature suggests that approximately 25% of addressees open a marketing email, while click through rates on banner ads are often in the 0.15% range [27, 28]. This suggests that our response rate may not be as poor as some may believe.

We offer two reasons why the results are credible despite the apparent low response rate. There is good congruence between the quantitative and qualitative results. As noted above, statements supportive of vaccines were frequent and are reflected by the high number of respondents who were vaccinated. Reasons to choose not to be vaccinated were less frequent than the reasons respondents chose in favor of vaccination. Self-reports of reasonable vaccine knowledge are supported by the use of credible vaccine resources. We would also note that many of our results are qualitative and, as such, are not intended to be generalizable to other populations. It seems reasonable to conclude that the results of the study are an accurate reflection of the opinions and behaviors of the respondents.

Although our objective was to study vaccine hesitancy in general, the results suggest that many of the responses address specific concerns about Covid-19 vaccines. This may introduce bias into the results, although it cannot be confirmed if the bias runs in favor of vaccines or against.

As in any survey, social desirability bias may be a concern. In this study, respondents were anonymous which may limit their concerns of having unpopular opinions become known. Additionally, the use of forced-choice responses can limit social desirability bias.

## Conclusions

Our results suggest that healthcare paraprofessionals are generally well informed about vaccines, have chosen to be vaccinated and use credible information resources when seeking vaccine information. However, much vaccine support was qualified and not all information sources were credible. This shows a need for continuing professional education for paraprofessionals. Such education should be designed to reduce fear and anxiety about vaccines.
